# In Vitro Assessment and Toxicological Prioritization of Pesticide Mixtures at Concentrations Derived from Real Exposure in Occupational Scenarios

**DOI:** 10.3390/ijerph19095202

**Published:** 2022-04-25

**Authors:** Sabrina Tait, Gabriele Lori, Roberta Tassinari, Cinzia La Rocca, Francesca Maranghi

**Affiliations:** 1 Center for Gender-Specific Medicine, Italian National Institute of Health, 00161 Rome, Italy or gabriele.lori@iss.it (G.L.); roberta.tassinari@iss.it (R.T.); cinzia.larocca@iss.it (C.L.R.); francesca.maranghi@iss.it (F.M.); 2 Science Department, Università Degli Studi di Roma Tre, Viale Guglielmo Marconi 446, 00146 Rome, Italy

**Keywords:** agrochemicals, mixtures, cumulative risk assessment, prioritization, ToxPI, cell death, oxidative stress, occupational exposure

## Abstract

Humans are daily exposed to multiple residues of pesticides with agricultural workers representing a subpopulation at higher risk. In this context, the cumulative risk assessment of pesticide mixtures is an urgent issue. The present study evaluated, as a case study, the toxicological profiles of thirteen pesticide mixtures used for grapevine protection, including ten active compounds (sulfur, potassium phosphonate, metrafenone, zoxamide, cyflufenamid, quinoxyfen, mancozeb, folpet, penconazole and dimethomorph), at concentrations used on field. A battery of in vitro tests for cell viability and oxidative stress endpoints (cytotoxicity, apoptosis, necrosis, ROS production, mitochondrial membrane potential, gene expression of markers for apoptosis and oxidative stress) was performed on two cellular models representative of main target organs of workers’ and population exposure: pulmonary A549 and hepatic HepG2 cell lines. All the endpoints provided evidence for effects also at the lower concentrations used. The overall data were integrated into the ToxPI tool obtaining a toxicity ranking of the mixtures, allowing to prioritize effects also among similarly composed blends. The clustering of the toxicological profiles further provided evidence of common and different modes of action of the mixtures. The approach demonstrated to be suitable for the purpose and it could be applied also in other contexts.

## 1. Introduction

Pesticides are man-made chemicals aimed to protect agricultural crops from pests, including insects, fungi and unwanted plants (weeds); they comprise insecticides, herbicides, fungicides and rodenticides. Although their use is strictly regulated [1], pesticides can be widely distributed in the environment, with potential harmful effects on humans. Indeed, several studies have reported the occurrence of their residues in a variety of matrices such as water, soil and food, as well as in outdoor and indoor air and house dust, confirming that the exposure is widespread and coming from a number of different sources [2,3,4].

Agricultural workers represent a subpopulation group at higher exposure, mainly by inhalation and dermal contact [5]. A recent review on health adverse effects pointed to an increased risk for some cancer types for this population group, with indications for DNA damage, oxidative stress and metabolic alterations [6]. Increased risk of neurodegenerative disorders such as Parkinson’s disease has been also reported [7,8]. In addition, thyroid derangements are more often associated to occupational exposure compared to non-occupationally exposed individuals [9].

Importantly, humans are simultaneously exposed to several pesticides [10,11,12], directly or as residues, in different combinations, whose joint/combined effects are still scarcely investigated [13]. Due to the relevance of such issue for human risk assessment, the European Commission (EC) and the European Food Safety Authority (EFSA) have recently published an action plan aimed to speed up the development of methods for the cumulative risk assessment (CRA) of pesticides for dietary and non-dietary exposures (https://ec.europa.eu/food/system/files/2021-03/pesticides_mrl_cum-risk-ass_action-plan.pdf; last access 28 February 2022).

It appears therefore clear that traditional toxicological studies performed on single chemicals are no longer suitable to fulfill this goal, and more complex approaches should be developed and implemented. In some studies, animals were exposed to real-life cocktails of pesticides and chemicals present in widely used consumer products, or as food additives, at concentrations close to the regulatory limits [14,15,16,17]. However, alternative and reliable methods to the use of animals are increasingly claimed among regulatory bodies, also to prioritize mixture toxicity [18]. Thus, new approach methodologies should be applied. For example, in the PERICLES project, combined effects of pesticides were assessed by firstly defining to which mixtures the general population was exposed through the diet, then investigating the effects of such mixtures by an in vitro approach [19].

In this framework, the aim of the present study is to evaluate and prioritize the toxicity of pesticide mixtures actually used on field by agricultural workers as a case study. Thirteen different mixtures used to treat and protect vineyards from the seasonal fungi and mold infections were indicated by Italian agronomists of the Italian autonomous Province of Trento (https://www.provincia.tn.it/; last access 5 April 2022). The active compounds included in the mixtures were: sulfur, generally used as a common component in most of the mixtures, potassium phosphonate, used in substitution or in combination with sulfur, and the fungicides metrafenone, cyflufenamid, quinoxyfen, folpet, penconazole, dimethomorph, mancozeb and zoxamide. Mixture toxicological profiles were assessed at concentrations used on field by a battery of assays aimed at discriminating potential different toxicity potency and focused on general toxicity, oxidative stress, and gene expression of markers for apoptosis (BAX, BCL2) and oxidative stress (NRF2). Genotoxicity evaluation was not included in the battery since all the single components of the mixtures were assessed to be not genotoxic in vivo via a relevant route of administration [20,21,22,23,24,25,26,27]; thus, the mixtures are also considered of no concern with respect to genotoxicity [28]. Human liver (HepG2) and lung (A549) cell lines were used as metabolically competent [29,30] and representative of the main target organs of the general population as well as occupational exposure. For dose-response curves, Benchmark Doses were calculated as more relevant for risk assessment [31]. The overall data were then integrated using the ToxPi tool to rank and prioritize mixture toxicity and to visualize similarities among the 13 toxicological profiles.

## 2. Materials and Methods

### 2.1. Chemicals and Mixtures

Sulfur (S, CAS N° 7704-34-9), metrafenone (MET, CAS N° 220899-03-6), cyflufenamid (CYF, CAS N° 180409-60-3), quinoxyfen (QUI, CAS N° 124495-18-7), folpet (FOL, CAS N° 133-07-3), penconazole (PEN, CAS N° 66246-88-6) and dimethomorph (DIM, CAS N° 110488-70-5) were dissolved in acetone, mancozeb (MAN, CAS no. 8018-01-7) and zoxamide (ZOX, CAS no. 156052-68-5) were dissolved in DMSO and potassium phosphonate (KP, CAS N° 13977-65-6) was dissolved in MilliQ water. Compounds and solvents were purchased from Sigma-Aldrich (Milan, Italy). Sterile working solutions were prepared just before use in culture medium.

Italian agronomists of the autonomous Province of Trento provided information on the composition of 13 mixtures used on field by agricultural workers, differently used according to season, in g/L of single active compounds. Such quantities were transformed into molar concentrations by dividing to the molecular weight of each pesticide. Mixture solutions were then prepared starting from field concentrations (and considered as 1:1 dilutions), as shown in Table 1, then performing 10-fold serial dilutions till 1:1,000,000 the field concentration. Mixture names are arbitrary. All the mixtures, except MIX7, contained S and three mixtures contained KP (i.e., MIX1, MIX4 and MIX7) as common components. Since S solubility is very low, the maximum achievable concentration in culture medium was 1:1000 the field concentration; thus, S was added at this dilution in 1:1 to 1:1000 mixtures, as shown underlined in Table 1. From 1:10,000 on, all the chemicals, including S, were at the same dilution. In addition, due to the limited solubility of QUI and MAN, 1:1 dilutions of the mixtures containing these compounds were not prepared to avoid overly acetone or DMSO concentrations in the culture medium, respectively.

### 2.2. Cell Cultures

Human hepatocellular carcinoma (HepG2) and lung carcinoma epithelial (A549) cell lines (ATCC, Manassas, VI, USA) were grown in DMEM without phenol red (Gibco, Milan, Italy), supplemented with 10% fetal bovine serum (Gibco), 100 U/mL penicillin, 100 µg/mL streptomycin (Gibco) and 2 mM L-Glutamine (Gibco). Cells were maintained in an incubator at 37 °C, 5% CO_2_ and 90% humidity.

### 2.3. Cytotoxicity Assays

Metabolic activity and cell proliferation of both HepG2 and A549 cells were assessed, respectively, by MTS (CellTiter 96^®^ AQueous One Solution reagent; Promega, Madison, WI, USA) and CyQUANT (CyQUANT^®^ Direct Cell Proliferation Assay; Life Technologies, Paisley, UK) assays by plating 10,000 cells/well on 96 flat-bottomed multiwells and incubating overnight at 37 °C. Medium was replaced with fresh medium containing ten-fold serial dilutions of the mixtures, as shown in Table 1, or vehicle as control at the percentage corresponding to the highest concentration tested for the chemicals, i.e., 1.2% acetone for QUI, 0.8% and 0.2% DMSO, respectively for MAN and ZOX. For all the other mixtures, maximum vehicle concentrations were below 0.1%. Each experimental point was assessed in triplicate. Plates were incubated for 24 h at 37 °C then adding 20 μL MTS reagent or 100 µL 2X CyQuant Detection Reagent to each well and further incubating for 1 h at 37 °C. Absorbance at 490 nm or green filter fluoresce (485 nm excitation–535 nm emission) were read by a Victor 3 Multilabel Reader (PerkinElmer, Waltham, MA, USA), for MTS and CyQuant assays, respectively, setting vehicle control cells as 100% viable. Each assay was repeated in three independent experiments.

Dose-response curves fitting was performed and visualized by GraphPad Prism v5.01 (GraphPad Software Inc., La Jolla, CA, USA), plotting log dilutions of field concentrations for each mixture. To derive relevant reference values for risk assessment [31], Benchmark Doses (BMD) were calculated by the EFSA web-tool for BMD analysis (https://r4eu.efsa.europa.eu, last access 23 November 2021), using as input log field dilutions of the mixtures. We set the Benchmark Response (BMR) equal to 10% mean change compared to controls and applied the model averaging, registering BMD_10_ lower and upper bounds (BMDL and BMDU) when a fitting was obtained.

### 2.4. Time Course Assessment of Apoptosis and Necrosis

The time course induction of apoptosis and necrosis by the pesticide mixture treatments was evaluated in HepG2 and A549 cells by the RealTime-Glo™ Annexin V Apoptosis and Necrosis Assay kit (Promega). 10,000 cells/well were plated on 96 white flat-bottomed multiwells, incubating overnight at 37 °C. Medium was then replaced with 1:10, 1:100 and 1:1000 dilutions of all the mixtures (Table 1) or with vehicles as control (acetone 1.2% or DMSO 0.8% and 0.2%) in duplicated wells, in presence of 100 μL/well of reagent mix solution. Plates were incubated at 37 °C reading both luminescence and fluorescence (485/535 nm) by the Victor 3 Multilabel Reader (PerkinElmer) after 5, 6, 7, 8 and 24 h, to detect apoptosis and necrosis signals, respectively. Fold change values with respect to vehicle control cells were calculated at each time for both endpoints. The assay was repeated in three independent experiments.

### 2.5. Reactive Oxygen Species (ROS) Intracellular Levels

Intracellular ROS levels were measured in HepG2 and A549 cells by the ROS Detection Assay Kit (BioVision, Milpitas, CA, USA). 10,000 cells/well were seeded on 96 flat-bottomed multiwells and incubated overnight at 37 °C. Medium was removed washing cells once with 100 μL ROS Assay Buffer, then incubating for 1 h at 37 °C with 100 μL/well 1X ROS Assay Label. After label solution removal, cells were treated for 24 h at 37 °C with 1:10, 1:100 and 1:1000 dilutions of all the mixtures (Table 1) or with vehicles as control (acetone 1.2% or DMSO 0.8% and 0.2%) in duplicated wells, or with H_2_O_2_ 100 μM as positive control. ROS production was detected by green fluorescence reading from the bottom (485/535 nm) by the Victor 3 Multilabel Reader (PerkinElmer), calculating fold change values compared to vehicle control cells after background subtraction. Three independent experiments were performed.

### 2.6. Mitochondrial Membrane Potential

The assessment of mitochondrial membrane potential collapse in HepG2 and A549 cells was performed by the Mitochondria Membrane Potential (MMP) Kit (Sigma-Aldrich). 10,000 cells/well were plated and incubated overnight at 37 °C; then medium was replaced with treatment medium containing 1:10, 1:100 and 1:1000 dilutions of all the mixtures (Table 1) or vehicles as control (acetone 1.2% or DMSO 0.8% and 0.2%) in duplicated wells. After 24 h at 37 °C, cells were added with 50 μL/well JC-10 Dye Loading Solution incubating 1 h at 37 °C. Reactions were stopped by adding 50 μL/well of assay buffer, then reading green fluorescence from the bottom to detect JC-10 monomers whose presence indicates MMP depolarization and failure to retain JC-10 aggregates. Fold change values compared to vehicle control cells were calculated; the assay was repeated in three independent experiments.

### 2.7. Gene Expression Analysis

HepG2 and A549 cells were plated with 300,000 cells/well in 6 flat-bottomed multiwells and incubated overnight at 37 °C. Medium was then removed and cells were treated for 24 h with 1:100 and 1:1000 dilutions (the first two non-cytotoxic) of all the mixtures (Table 1) or medium alone as control. At the end, cells were harvested and centrifuged storing cell pellets at –80 °C until analysis. Three independent treatments were performed at different cell passages. Total RNA was extracted from each sample by the Norgen RNA Kit (Norgen, Thorold, Canada), assessing RNA quantity with a Nabi Nano Spectrophotometer (MicroDigital Co. Ltd., Korea). For each sample, 1 μg of total RNA was reverse- transcribed to cDNA by the SensiFast™ cDNA Synthesis Kit (Bioline Reagents Ltd., London, UK). Specific primers for BCL2 apoptosis regulator (BCL2), BCL2 associated X apoptosis regulator (BAX), nuclear factor erythroid 2 like 2 (NRF2), as well as for TATA-box binding protein (TBP) as reference gene (listed in Table 2) were designed with Primer-BLAST (www.ncbi.nlm.nih.gov/tools/primer-blast, last access 9 November 2020) and purchased from Invitrogen (Thermo Fisher Scientific). PCR reactions were prepared with the Excel Taq^TM^ Fast Q-PCR Master Mix SYBR (SMOBIO Technology Inc., Hsinchu City, Taiwan) and run in duplicate in a Bioer LineGene 9600 Plus thermocycler instrument (Bioer Technology Co. Ltd., Hangzhou, China) with the following thermal program: 1 cycle at 95 °C for 20 s; 40 cycles at 95 °C for 3 s, 58 °C for 15 s and 72 °C for 15 s; 1 melting cycle from 55 to 95 °C, 30 s/°C to verify amplification products. Threshold cycles (Ct) in each condition were identified by the LineGene 9600 PCR V.1.0 software (Bioer), then calculating ΔΔCt values, with control cells as calibrators and TBP as normalizer.

### 2.8. ToxPi Score Calculation

The ToxPi v2.3 Graphical User Interface [32] was used to derive relative toxicological indexes and prioritize mixtures, as previously described [33]. ToxPi scores are dimensionless normalized values between 0 and 1, displayed as a pie with each slice representing an endpoint: the larger the radius of a slice, the greater the effect. Briefly, data were divided into two domains, Assays and Pathways; the first comprised cytotoxicity, apoptosis, necrosis, ROS and MMP assays data, whereas Pathways included gene expression data. In particular, for cytotoxicity, the BMD_10_ values for each mixture in metabolic activity and cell proliferation assays, as log dilution of field concentrations, were included (Slice 1), as well as maximum effect levels observed at 1:10 field dilutions (Slice 2), being the first dilution common to all mixtures. For ROS and MMP data, mean fold change values obtained in the assays at the three tested dilutions (i.e., 1:10, 1:100 and 1:1000) were considered (Slices 3 and 4, respectively), and for apoptosis/necrosis data, mean fold change values at 8 and 24 h for the three tested dilutions were included (Slices 6 and 7). Finally, in the Pathways domain, mean ΔΔCt values for each gene at each tested dilution were included (Slice 8). Percent, fold change and ΔΔCt values were expressed as absolute difference values from 100%, 1-fold or zero, respectively, to take into account both the effects of up- and downregulation. Log dilution values were scaled to –log10(x) values, whereas all the other values were not scaled and left in linear scale. All slices were equally weighted to perform the toxicological scores’ calculation. The hierarchical clustering was also visualized to compare similar toxicological profiles.

### 2.9. Statistical Analysis

The JMP 10 software (SAS Institute srl, Milan, Italy) was used to assess significant differences between treated and control groups by performing one-way analysis of variance (ANOVA) with post-hoc Dunnett’s test where applicable, setting significance at *p*-value < 0.05.

## 3. Results

### 3.1. Cytotoxicity

**Figure 1:**
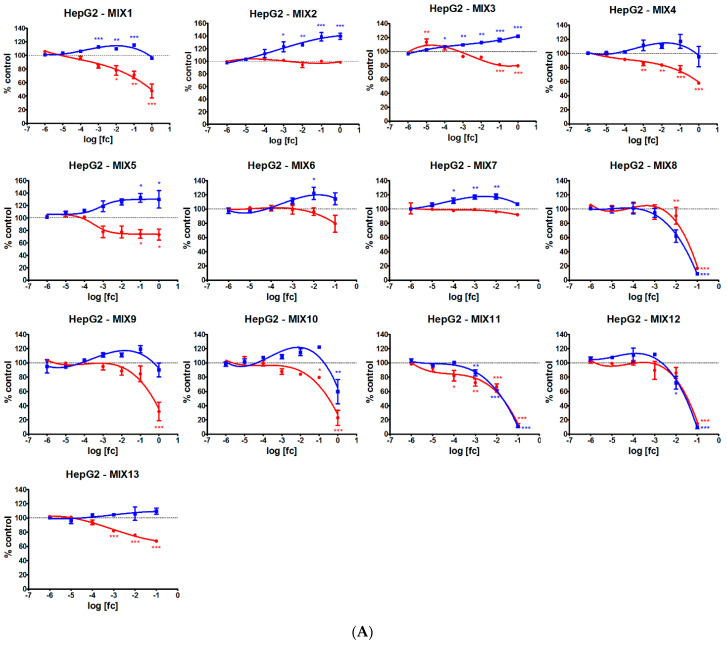

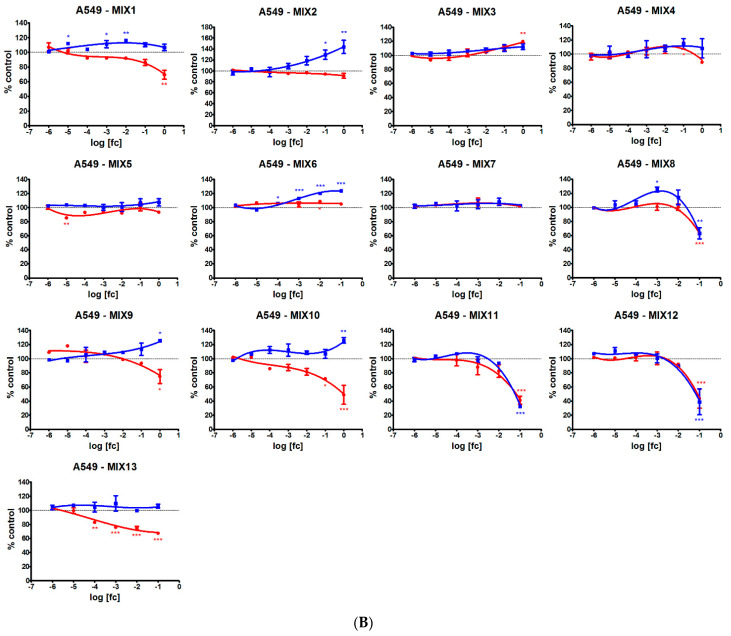
Dose-response curves related to MTS (blue lines) and CyQuant (red lines) assays in (**A**) HepG2 and (**B**) A549 cell lines treated with the 13 pesticide mixtures for 24 h. Data represent mean absorbance (MTS) and fluorescence (CyQuant) signals of three independent experiments normalized to control cells set as 100%. Concentrations are expressed as log of the field concentration [fc] dilution applied. Asterisks indicate the level of significance: * *p* < 0.05; ** *p* < 0.01; *** *p* < 0.001.

The 13 mixtures differently affected the metabolic activity and cell proliferation of HepG2 and A549 cells (Figure 1). In HepG2, mixtures containing MET and/or ZOX (as MIX1, MIX3 and MIX5) induced a significant dose-dependent increase in metabolic activity while they reduced cell proliferation; a similar trend was noted also in MIX4, although the metabolic activity induction was not significant. Among these mixtures, only MIX1 exerted a similar effect also in A549 cells whereas the other had no significant effect or slightly increased cell proliferation at 1:10 field dilution (i.e., MIX3). The other mixture with MET, MIX2, did not affect cell proliferation but induced a significant dose-related increase of metabolic activity in both cell lines.

Two of the mixtures featuring QUI (MIX6 and MIX7) induced metabolic activity in HepG2 but did not affect cell proliferation; in A549, only MIX6 determined the same pattern, whereas MIX7 had no effect. MIX13, containing QUI and DIM, dose-dependently decreased cell proliferation but did not affect metabolic activity in both cell lines. Mixtures containing FOL (MIX9 and MIX10) were cytotoxic at field concentrations, reducing cell proliferation by about 70 and 77% in HepG2 and by about 25 and 51% in A549, respectively, for MIX9 and MIX10. At the same field concentrations, MIX10 decreased metabolic activity by about 40% in HepG2 but both MIX9 and MIX10 increased it in A549 by about 25%.

The most cytotoxic mixtures were those featuring MAN which, at 1:10 field concentrations, severely reduced metabolic activity and cell proliferation in both cell lines. In HepG2, effects were even evident from 1:100 dilution in cells treated with MIX8 and MIX12 and from 1:10,000 dilution in cells treated with MIX11.

Table 3 shows the BMD_10_ values calculated for all the dose-response curves in the two cell lines. Values in HepG2 cells are generally lower than in A549 cells, supporting a higher responsiveness of hepatic cells to pesticide exposure effects.

### 3.2. Apoptosis/Necrosis

**Figure 2:**
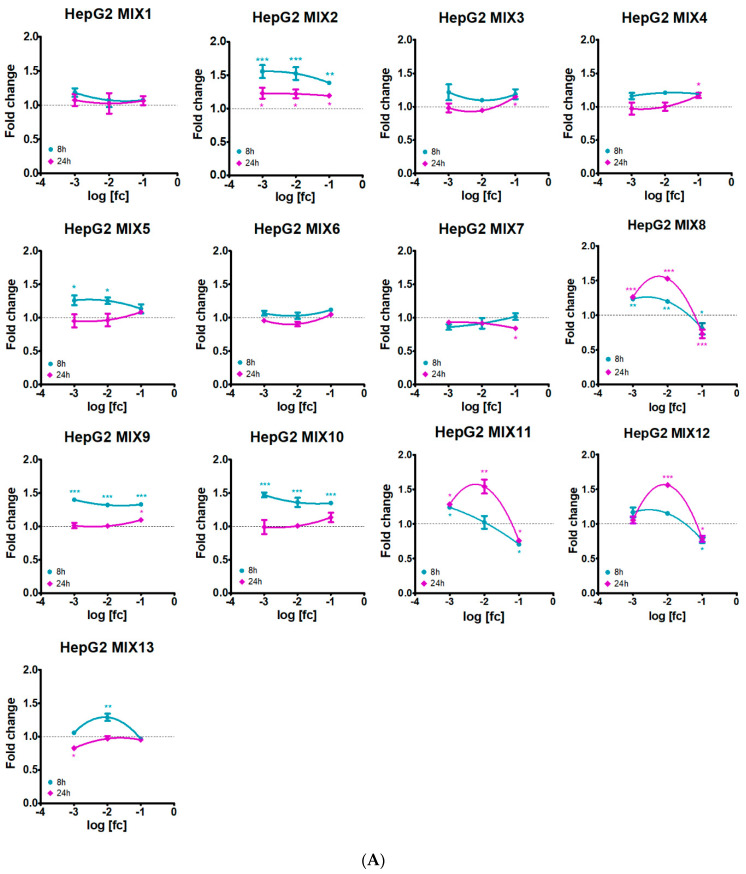

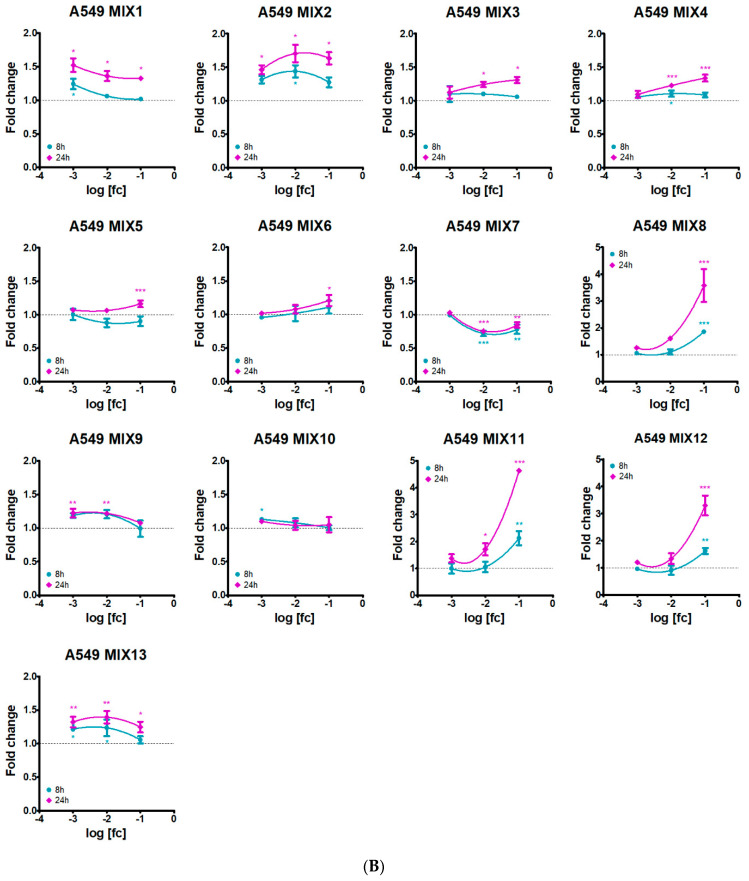
Dose-response curves of apoptosis assessment in (**A**) HepG2 and (**B**) A549 cell lines treated with the 13 pesticide mixtures for 8 h (dark turquoise lines) and 24 h (dark magenta lines). Concentrations are expressed as log of the field concentration [fc] dilution applied. Data represent mean fold change luminescence signals, compared to control cells, of three independent experiments. Asterisks indicate the level of significance: * *p* < 0.05; ** *p* < 0.01; *** *p* < 0.001.

**Figure 3:**
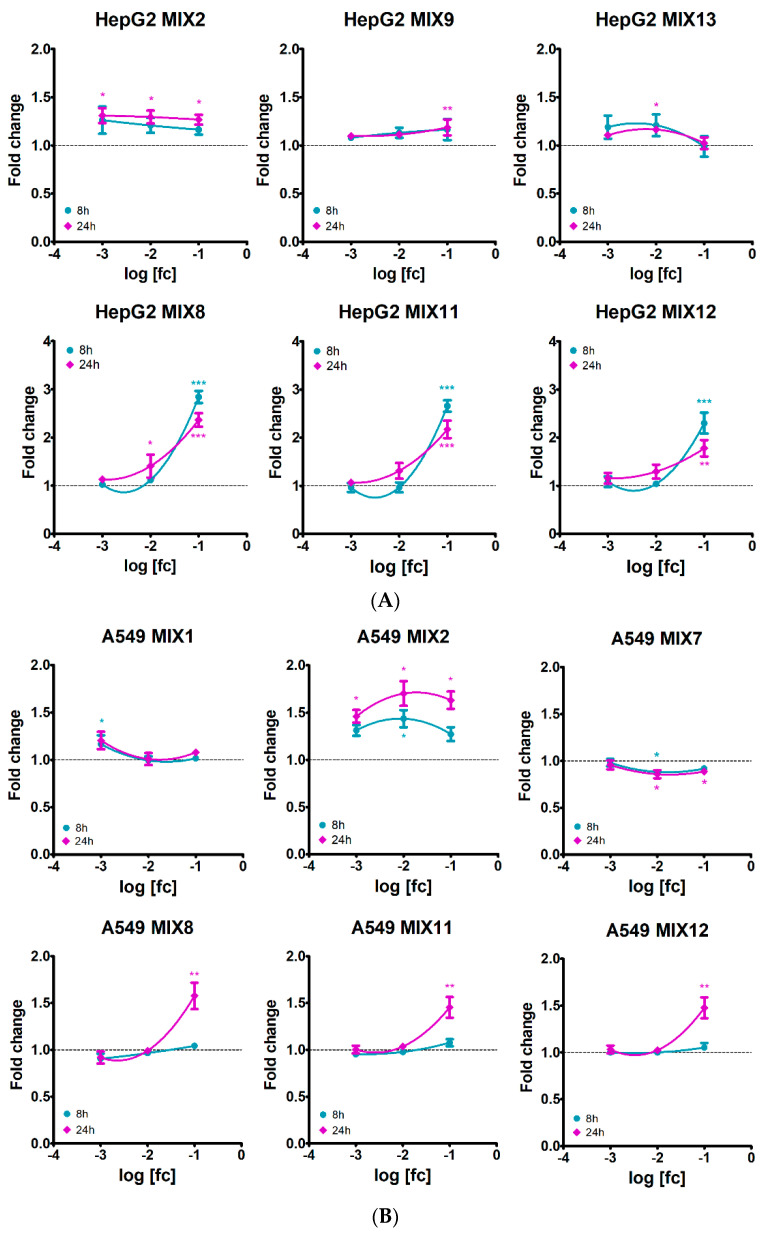
Dose-response curves of necrosis assessment in (**A**) HepG2 and (**B**) A549 cell lines treated with different pesticide mixtures for 8 h (dark turquoise lines) and 24 h (dark magenta lines). Concentrations are expressed as log of the field concentration [fc] dilution applied. Data represent mean fold change fluorescence signals, compared to control cells, of three independent experiments. Among the 13 pesticide mixtures tested, only graphs with significant effects are shown. Asterisks indicate the level of significance: * *p* < 0.05; ** *p* < 0.01; *** *p* < 0.001.

Different induction of apoptosis and necrosis processes was observed following exposure to the 13 mixtures in the two cell lines, but with some similarities among mixtures with common compounds. Figure 2 and Figure 3 show dose-response curves related to apoptosis and necrosis after 8 h and 24 h treatment. Complete time-courses from 5 h are available as Supplementary Figures S1 and S2. In HepG2 cells (Figure 2A), a higher apoptotic effect at 8h, then declining at 24h, was generally observed, especially in the mixtures containing MET (MIX2), QUI (MIX13) and FOL (MIX9 and MIX10). Interestingly, the three mixtures containing QUI behaved differently according to the field dilution (Figure 2A and Supplementary Figure S1); indeed, MIX6 did not affect apoptosis at all tested dilutions, whereas MIX7 decreased the apoptotic signal compared to control cells in 1:100 and 1:1000 dilutions, also evident for 1:10 dilution at 24 h; MIX13 induced apoptosis till 8 h only at 1:100 dilution.

MIX3, MIX4 and MIX5, all featuring ZOX, determined a constant increase in apoptosis till 24 h at 1:10 field dilution (significant only for MIX3 and MIX4), whereas 1:100 and 1:1000 dilutions induced apoptosis till 8 h.

In HepG2 cells treated with MIX8, MIX11 and MIX12, all featuring MAN, the 1:10 field concentration exerted a time-dependent decrease in the apoptotic signal, most probably due to the strong cytotoxicity induced by these mixtures and the consequent decrease in cell number. The 1:1000 dilution of the three mixtures constantly increased apoptosis over time, except for MIX12, declining from 7 h on. The most striking effect was observed for 1:100 dilutions of these mixtures, all determining a strong increase in apoptosis at 24 h, and differing for no response till 8 h (MIX11 and MIX12) or induction starting from 7 h on (MIX8).

In A549 cells (Figure 2B and Supplementary Figure S1), almost all the mixtures induced apoptosis at 24 h, especially at 1:10 field dilution. As observed in HepG2 cells, 1:10 dilution of the mixtures with MAN (MIX8, MIX11 and MIX12) strongly activated apoptosis at 24 h, to a higher extent than that observed in hepatic cells. For some mixtures, the 1:10 dilution was the only one to significantly increase apoptosis (MIX5, MIX6, MIX8, MIX12), whereas for other mixtures also 1:100 and 1:1000 dilutions promoted this process (MIX1, MIX2, MIX3, MIX4, MIX9, MIX11, MIX13), in some cases with the effect starting earlier (MIX2 and MIX13). Interestingly, 1:10 and 1:100 dilutions of MIX7 drove a drop in apoptosis compared to control cells.

Activation of the apoptotic event did not always lead to necrosis, so only significant necrotic effects are shown in Figure 3 and Supplementary Figure S2. A slightly significant increase in the necrotic signal was observed in cells treated with MIX2 (featuring MET), at all the three dilutions, at 24 h, in hepatic cells and at 1:10 and 1:100 dilutions in lung cells, with also some earlier effects. Another mixture with MET, MIX1, induced some necrosis only in A549 cells, almost constantly across time, at 1:1000 dilution.

In HepG2, MIX9 (featuring FOL) induced cell necrosis only at 1:10 dilution at 24 h, whereas MIX13 (featuring QUI and DIM) determined an increase in necrosis at an earlier time at 1:100 and 1:1000 dilutions, slightly declining at 24 h. As observed also for apoptosis, MIX7 determined a drop in the necrosis signal compared to control A549 cells at 1:10 and 1:100 dilutions, over time.

Similarly to apoptosis, the more remarkable effect on necrosis was stimulated by the 1:10 dilution of the three mixtures containing MAN (MIX8, MIX11 and MIX12) which greatly induced necrosis in HepG2 from 5 h on, declining at 24 h. Only for MIX8, also the 1:100 dilution significantly increased necrosis at 24 h. In A549, the 1:10 dilution of these three mixtures promoted necrosis only at 24 h.

### 3.3. ROS Levels

**Figure 4:**
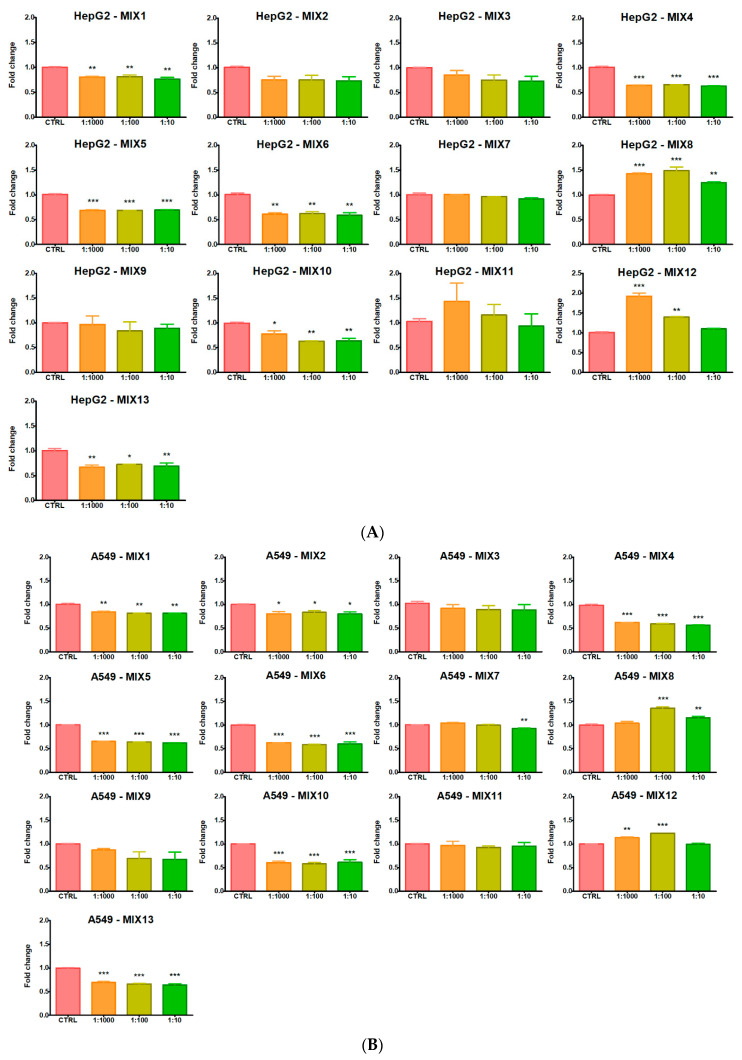
Intracellular ROS levels in (**A**) HepG2 and (**B**) A549 cell lines treated with the 13 pesticide mixtures for 24 h at 1:10, 1:100 and 1:1000 field concentrations. Data represent mean fold change fluorescence signals, compared to control cells, of three independent experiments. Asterisks indicate the level of significance: * *p* < 0.05; ** *p* < 0.01; *** *p* < 0.001.

Each of the 13 mixtures similarly affected ROS intracellular levels in HepG2 and A549 cells (Figure 4). Notably, after treatment with MIX1, MIX4, MIX5, MIX6, MIX10 and MIX13, both HepG2 and A549 cells showed decreased intracellular ROS levels compared to control cells, almost to the same extent at all three dilution conditions. MIX2 significantly exerted the same effect only in A549 cells, although a decrease at all dilutions was noted also in HepG2. MIX3, MIX9 and MIX11 did not significantly alter ROS levels in both cell lines. Conversely to MIX11, the other two mixtures containing MAN (MIX8 and MIX12) significantly induced ROS levels in both cell lines but with different patterns: MIX8 at all three dilutions in HepG2, and at 1:10 and 1:100 dilutions in A549 cells; MIX12 at 1:100 and 1:1000 dilutions in both cells. Lastly, MIX7 reduced ROS levels at 1:10 dilution in A549 cells.

The positive control H_2_O_2_ greatly increased ROS levels, as expected (data not shown).

### 3.4. Mitochondrial Membrane Potential

**Figure 5:**
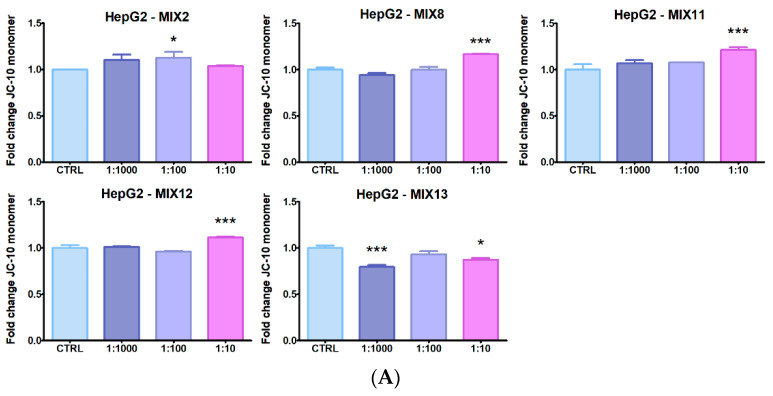

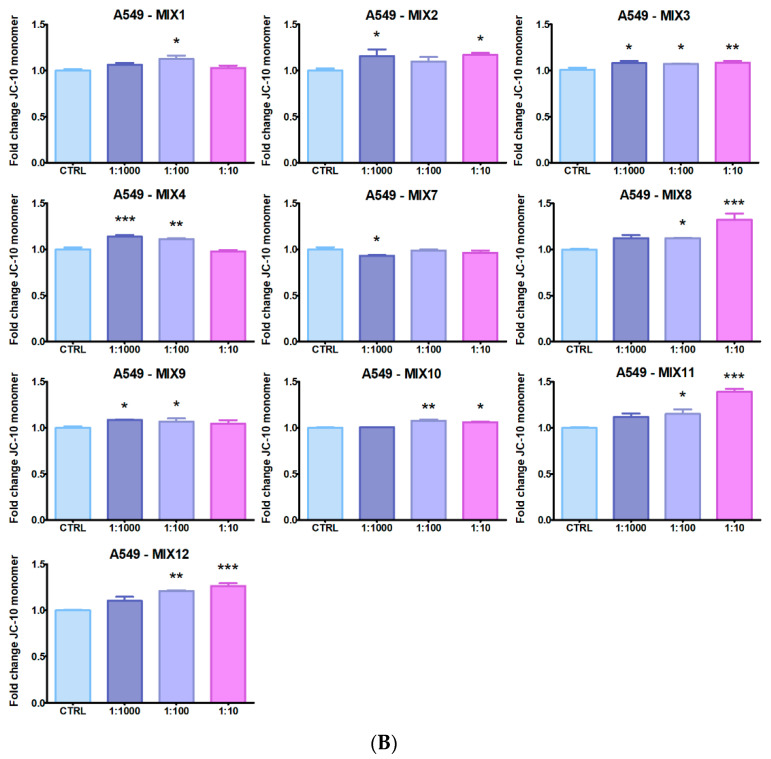
JC-10 monomer abundance, indicating collapse of the mitochondrial membrane potential, in (**A**) HepG2 and (**B**) A549 cell lines treated with the 13 pesticide mixtures for 24 h at 1:10, 1:100 and 1:1000 field concentrations. Data represent mean fold change fluorescence signals, compared to control cells, of three independent experiments. Only graphs with significant effects are shown. Asterisks indicate the level of significance: * *p* < 0.05; ** *p* < 0.01; *** *p* < 0.001.

In HepG2 cells (Figure 5A), the only mixtures impairing mitochondrial membrane potential (MMP) were those containing MAN (MIX8, MIX11 and MIX12) at 1:10 dilutions. Also MIX2, featuring MET, determined a decrease in MMP at 1:100 dilution. On the contrary, MIX13 (with QUI and DIM) increased MMP at 1:10 and 1:1000 dilutions.

Effects on A549 cells were more pronounced (Figure 5B), with MIX1, MIX2 and MIX3 (featuring MET), MIX4 (with ZOX + CYF), MIX9 and MIX10 (with FOL) and MIX8, MIX11 and MIX12 (with MAN), all impairing MMP, at different dilutions according to the mixtures, but with more striking effects following treatment with 1:10 dilutions of MAN-containing mixtures. Only MIX7 (with QUI) increased MMP at the highest dilution.

### 3.5. Real-Time PCR

**Figure 6:**
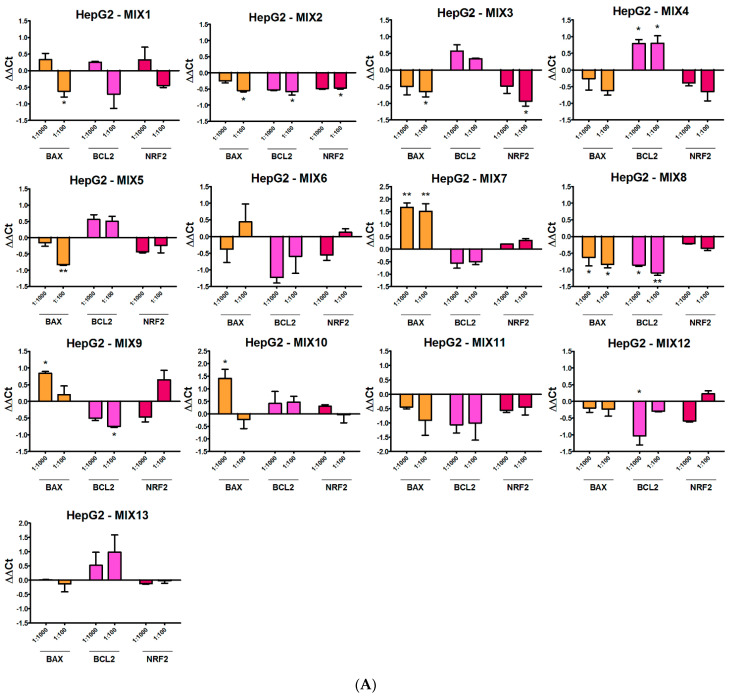

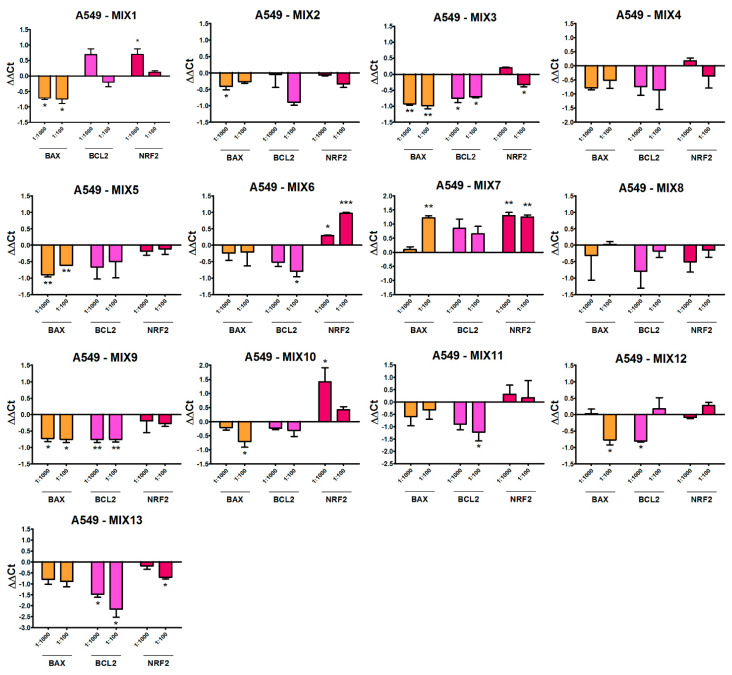
Expression of BAX, BCL2 and NRF2 genes in (**A**) HepG2 and (**B**) A549 cell lines treated with the 13 pesticide mixtures for 24 h at 1:100 and 1:1000 field concentrations. Data represent mean ΔΔCt values of three independent experiments, with control cells as calibrator and TBP gene as normalizer. Asterisks indicate the level of significance: * *p* < 0.05; ** *p* < 0.01; *** *p* < 0.001.

Assessment of the expression of apoptotic (BAX and BCL2) and oxidative stress (NRF2) genes at non-toxic concentrations (1:100 and 1:1000 dilutions) further supported the different actions of the mixtures under study (Figure 6). Mixtures featuring MET and/or ZOX (MIX1, MIX2, MIX3 and MIX5) significantly repressed BAX expression in HepG2 at the 1:100 dilution. Such mixtures had comparable effects in A549 cells, sometimes driving a significant inhibition also at the 1:1000 dilution. MIX4, also featuring ZOX, displayed a similar but not significant pattern. Among the mixtures containing QUI, MIX6 and MIX13 did not affect BAX expression in both HepG2 and A549 cells, whereas MIX7 strongly induced it at both dilutions in HepG2 and at 1:100 dilution in A549. Interestingly, the mixtures containing FOL (MIX9 and MIX10) induced BAX expression in HepG2 cells at the 1:1000 dilution, whereas they repressed it in A549. MIX8 was the only MAN-containing mixture repressing BAX, at both dilutions, in HepG2 cells only; MIX11 did not alter its expression in both cell lines, while MIX12 induced a decline in A549 cells at 1:100 dilution.

The anti-apoptotic BCL2 gene was significantly induced only by MIX4 (with ZOX+ CYF) in HepG2 cells. Conversely, MIX2 (with MET), MIX8 and MIX12 (with MAN) and MIX9 (with FOL) all repressed BCL2 expression in hepatic cells, at both or only one dilution. In A549, no mixture induced BCL2 expression, whereas MIX3 (with MET + ZOX), MIX6 and MIX13 (with QUI), MIX9 (with FOL), MIX11 and MIX12 (with MAN) inhibited its expression, at one or both dilutions.

In HepG2 cells, no induction of the NRF2 gene was observed; rather, MIX2 and MIX3 (with MET) repressed its expression at the 1:100 dilution. Conversely, in A549, MIX1 (with MET), MIX6 and MIX7 (with QUI) and MIX10 (with FOL and PEN), increased NRF2 gene expression. MIX3 (with MET +ZOX) and MIX13 (with QUI + DIM) down-regulated NRF2 expression in A549 cells.

### 3.6. ToxPi Score

The calculation of the cumulative toxicological scores for the 13 mixtures yielded the three containing MAN (MIX11, MIX8 and MIX12) as the more toxic (Table 4), followed by MIX2, containing MET, and MIX13 and MIX6, containing QUI. The next three mixtures all featured ZOX (i.e., MIX3, MIX4 and MIX5), then MIX10 and MIX9, containing FOL, MIX7 with QUI and lastly MIX1 with MET. Comparing the ToxPi scores among mixtures with common pesticides, it is noteworthy that those containing PEN are more toxic than those without, as for MIX11 > MIX8 and MIX10 > MIX9. On the contrary, mixtures containing KP are less toxic, as for MIX7 < MIX6, in which KP and S are alternatively present, and MIX1 < MIX2. The same does not occur for MIX4 and MIX5, but these mixtures feature also CYF. Interestingly, MET alone (MIX2) was more toxic than in combination with ZOX (MIX3). Conversely, MET with MAN (MIX12) was less toxic than MAN alone (MIX8). Moreover, DIM in combination with QUI increased toxicity (MIX13) compared to QUI alone (MIX6 and MIX7).

Toxicity scoring in each cell line slightly differed from the overall score; of note is the different MIX5 toxicity, much higher in HepG2 than in A549 cells (at 6th and 12th ranking position, respectively), and the higher toxicity of MIX2, second in the ranking, and of FOL-containing mixtures (MIX10 and MIX9) in A549 compared to HepG2 cells.

**Figure 7:**
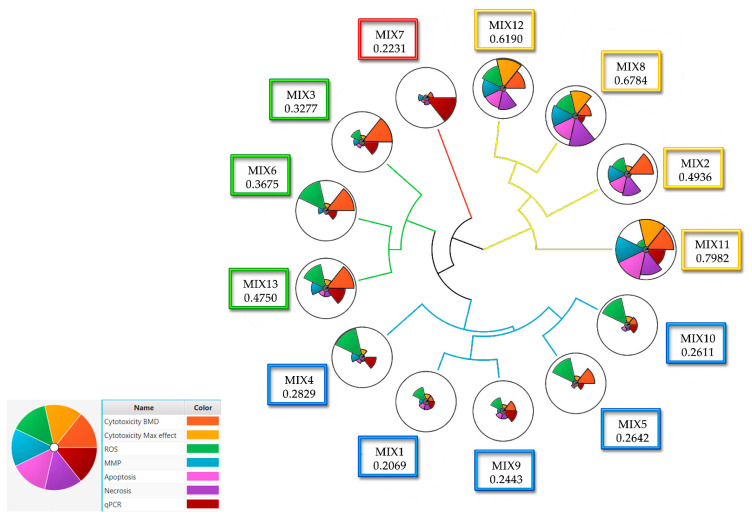
Hierarchical clustering of the ToxPi profiles of the 13 pesticide mixtures on the basis of the overall toxicological data evidenced by the connecting yellow, red, green and cyan lines. Corresponding ToxPi scores for the total calculation are indicated in each box.

The hierarchical clustering obtained with ToxPI (Figure 7) evidenced toxicological profile similarities among mixtures, identifying four different clusters. In the cluster with the three mixtures featuring MAN (MIX8, MIX11 and MIX12), there was also MIX2 with MET (yellow connecting lines), and all shared relevant contributions from BMD_10_ Cytotoxicity, MMP, Apoptosis and Necrosis slices. The second cluster (green lines) included MIX13 and MIX6 (with QUI) and MIX3 (with MET+ ZOX), for which the most contributing slices were BMD_10_ Cytotoxicity, followed by ROS and qPCR. The largest cluster (cyan lines) featured mixtures (MIX4 and MIX5 with ZOX; MIX9 and MIX10 with FOL; MIX1 with MET) whose toxicity mostly derived from the ROS slice. The last cluster (red line) only featured MIX7 (with QUI), whose main contribution to toxicity came from qPCR.

## 4. Discussion

The assessment of the risks posed by pesticides in mixtures, as happens in real exposure scenarios, has gained growing attention during the last decade, especially for agricultural worker’s safety [6,34]. Indeed, the regulatory evaluation of single compounds does not guarantee the protection from additive or synergistic effects, especially when modes of action or targets overlap [35].

In this context, the present study provided an approach to compare the toxicological responses induced by 13 pesticide mixtures actually used for grapevine protection, at concentrations used on field by agricultural workers. Ten active compounds were differently combined in the 13 mixtures. A battery of assays was selected to evidence different modes of action, especially related to oxidative stress, on two in vitro models representative of main target organs. The overall data were integrated using the ToxPI tool which ranked and clustered the toxicological profiles of the 13 mixtures. All the selected assays provided relevant information contributing to the toxicological profiles, thus allowing to discriminate potency also among similar mixtures.

In general, the most abundant active compounds ‘weighed’ more on the toxicological profiles, but minor components, such as PEN and CYF, used at concentrations an order of magnitude lower, somehow influenced the final toxicity outcomes. Moreover, it can be excluded that the presence of inorganic S and KP, widely used as active ingredients for grapevine treatment, could have contributed to any toxicological effect since both have been assessed as being scarsely toxic [36,37]. Rather, generally, toxic effects were decreased in presence of KP, as described below.

Among the real pesticide mixtures under study, those containing MAN (MIX8, MIX11 and MIX12) had the highest ToxPi scores, exerting severe cytotoxic effects at 1:10 dilution by a mechanism involving early apoptosis and secondary necrosis, especially in HepG2 cells, where necrosis was evident from 5 h after the treatment. MIX8 and MIX12 caused a significant induction in ROS intracellular levels, and all three MAN-containing mixtures impaired MMP in both cell lines, especially at 1:10 dilution. More relevant effects on gene expression were observed upon treatment with MIX8 (S + MAN). Compared to MIX8, the addition of MET (MIX12) determined a lower ToxPI score, whereas PEN increased the toxicity (MIX11).

Interestingly, MIX2 (S + MET) is in the same cluster of MAN-containing mixtures. More peculiarly, the mixtures containing MET did not cluster together but in three different clusters. Thus, it can be hypothesized that MET differently interacts with other components in the mixtures. MIX2 was the only mixture affecting almost all the endpoints under study; although not altering cell proliferation, MIX2 dose-dependently increased metabolic activity and more severely affected apoptosis, also at earlier time points, and necrosis. Further, MIX2 decreased ROS levels in HepG2 cells and impaired MMP of both cell lines. The additional presence of ZOX in MIX3 (S + MET + ZOX), partially decreased the overall toxicity, even if it repressed cell proliferation in HepG2; in particular, apoptosis was evident only at 24 h at 1:10 dilution, with no concomitant necrotic effect. Moreover, MIX3 did not affect ROS levels and impaired MMP only in A549 cells. The pesticide combination in MIX1 (S + KP + MET) further decreased the ToxPI score, but, conversely to MIX3, this mixture affected all the endpoints, but to a minor extent with respect to MIX2. Indeed, MIX1 decreased cell proliferation in both cell lines and exerted some apoptotic and necrotic effects, although only in A549 at the highest dilution tested (1:1000); moreover, it decreased ROS levels in both cell lines and impaired MMP in A549 cells. Effects on the gene expression were more evident for MIX2 in HepG2 cells and for MIX3 in A549 cells.

In the second cluster for toxic potency, besides MIX3, two mixtures featuring QUI (MIX6 and MIX13) were present. The other mixture with QUI, MIX7, containing KP instead of S, was less toxic and clustered alone. Thus, similarly to what was observed for MIX2 and MIX1, the presence of KP lowered the ToxPI score. It would be interesting to further investigate which underling mechanisms determine the KP antagonizing effect. Indeed, compared to the other QUI-containing mixtures, MIX7 only marginally increased the metabolic activity of HepG2 cells and determined a singular decrease of the apoptotic and necrotic signals. It slightly affected ROS and MMP in A549 cells; however, it increased BAX expression in both cells lines and NRF2 in A549 cells.

MIX13 (S + QUI + DIM), was more toxic than MIX6 (S + QUI). Indeed, MIX13 more severely affected cell proliferation and increased apoptosis in both cell lines and necrosis in HepG2, also at earlier time points. Both mixtures decreased ROS levels in HepG2 and A549 cells but only MIX13 compromised MMP in HepG2; thus, an additional contribution of DIM to toxicity could be hypothesized. None of the two mixtures affected gene expression in HepG2 cells and they differently modulated BCL2 and NRF2 in A549.

The last cluster featured mixtures containing ZOX (MIX4 and MIX5), FOL (MIX9 and MIX10) and MIX1 with MET, already described. Compared to MIX3 (S + MET + ZOX), MIX4 (S + PK + ZOX + CYF) and MIX5 (S + ZOX + CYF) were less toxic, suggesting an antagonizing effect of CYF; however, they had cytotoxic profiles similar to MIX3, but they increased apoptosis in HepG2 cells at earlier time points, and in A549 at 24 h with no effect on necrosis. Both MIX4 and MIX5 decreased ROS levels in both cells but only MIX4 impaired MMP in A549 and increased BCL2 in HepG2, while MIX5 decreased BAX in both cell lines.

Considering the two mixtures with FOL, MIX9 (S + FOL) and MIX10 (S + FOL + PEN), PEN increased the ToxPI score, similarly to what was observed in combination with MAN (MIX11). The two mixtures had comparable cytotoxic profiles, similarly induced apoptosis in both cell lines, compromised MMP in A549 cells and affected gene expression, whereas only MIX9 increased necrosis at 24 h in HepG2 cells and MIX10 highly decreased ROS levels in both cell lines.

Among the active compounds included in the 13 mixtures, MAN is certainly the most toxic. MAN is an ethylene bis-dithiocarbamate fungicide whose toxicity is due to the main metabolite ethylenethiourea, affecting thyroid functionality in animal studies [27]. We previously observed similar effects with MAN alone in HepG2 and A549 cells were in the same range of concentrations as in the present study [38]. Other in vitro studies further support the observed increase in cytotoxicity, apoptosis, ROS production and mitochondrial impairment, as evidenced in human and mouse cell lines [39,40,41]. The present data provided further evidence for a severe necrotic effect induced by MAN-containing mixtures, independently from other co-present active compounds.

We previously assessed also ZOX toxicity in HepG2 and A549 cells in the same range of concentrations and with similar effects as in the present study on most of the endpoints [38]. The only other available in vitro study showed an IC_50_ of 30 nM for cytotoxicity in Chinese hamster fibroblasts [42]. In addition, due to ZOX’s inhibiting action on tubulin polymerization, detrimental effects on development, oxidative stress balance and apoptosis were observed in zebrafish embryos [43], supporting the present observation on cell death and MMP impairment.

Limited evidence is available for MET effects. Only one study on mouse cortical neurons showed cytotoxicity after 7 days of exposure, and a slight mitochondrial membrane depolarization [44], in line with the present data on MET-containing mixtures.

To our knowledge, the only available toxicological assessment of QUI demonstrated its aryl hydrocarbon receptor agonistic effects in transfected HepG2 cells and the concomitant induction of zebrafish recombinant *CYP1A1* gene expression [45]; this evidence substantiates the present observation of increased NRF2 expression induced by QUI mixtures (MIX6 and MIX7) in A549 cells.

FOL is a fungicide inducing cancer in the mouse gastrointestinal tract, especially the duodenum [24]. Previous evidence on FOL pointed to induction of cell death, disruption of mitochondria potential and increase in ROS production in mouse Sertoli cells [46]. A FOL formulation exerted cytotoxic effects on human bronchial epithelial cells associated with apoptosis, necrosis and increased oxidative stress with ROS production [47], all confirming the present results, except for ROS, which we found decreased upon FOL-containing mixture exposure.

PEN is a triazole fungicide that was demonstrated to damage lung histology by increasing oxidative stress in adult rats [48], and which displayed (neuro)developmental toxicity in zebrafish [49,50]. Cytotoxic effects were observed in T-47D thyroid cells, whose survival was decreased by about 15% following treatment with 10 µM PEN [51]. The results of the present study indicate that PEN may display some agonistic effects increasing the ToxPI score of the mixtures.

The only available evidence on CYF toxicological assessment was included in the EFSA report, showing increased incidence of thyroid and hepatic carcinomas in rats and mice, respectively [23]. No in vitro studies are available to be compared with the present data. The results of this study included the CYF-mixtures in the lowest toxicological cluster.

According to an EFSA report, DIM showed limited toxicity in the liver, testes and prostate of dogs in a one-year study [21]. In the only in vitro report, DIM decreased cell viability of mouse cortical neurons but did not affect MMP [44]; thus, it is reasonable to hypothesize that DIM had an additive effect with QUI on cytotoxicity, increasing also apoptosis.

Among the endpoints assessed in the present study, the more intriguing to be further and more deeply investigated is the decrease in ROS intracellular levels exerted by the majority of mixtures, which allows to speculate an autophagy mechanism [52].

These results clearly demonstrate that the toxicological assessment of pesticide mixtures used on field—representing a real exposure scenario—can be accomplished also when minimum information is available on the single active compounds, since the ToxPI tool, by the integration of multiple sources of data and reduction of variables to a single dimensionless score, is able to capture also minor differences between similar mixtures. Such approach could be easily extended to other real-life mixtures, with a higher number of endpoints and cellular models implied, to provide robust data for cumulative risk assessment and mixture prioritization, especially in occupational contexts, to guide the choice of mixtures with a greater safety margin of exposure.

## 5. Conclusions

The present approach demonstrated to be a valuable tool to support the prioritization of commercial chemical mixtures or mixtures for which not all the components are completely characterized, by comparing their toxicological profile and ranking their scores. The data provided by the battery of in vitro tests and integrated into the ToxPI tool, highlighted that mixtures containing MAN were the more toxic, providing further supporting evidence for MET’s toxicity. Differences among similar mixtures were also caught, thus supporting the reliability of the approach, which could be implemented including more and different endpoints. The application of such an approach in other cumulative risk assessment areas could provide relevant information to reduce health risks either for the general population and for professional exposure such as agricultural workers.

## Figures and Tables

**Table 1:** Composition of the 13 pesticide mixtures. Serial dilutions of field concentrations and corresponding nominal concentrations of each active compound are indicated.

MIXTURES	1X 1:1 (FIELD CONC.)	1E-1X 1:10	1E-2X (1:100)	1E-3X (1:1000)	1E-4X (1:10,000)	1E-5X (1:100,000)	1E-6 (1:1,000,000)
**MIX1**	S (124.7 µM) PK (18.9 mM) MET (305.4 µM)	S (124.7 µM) PK (1.89 mM) MET (30.54 µM)	S (124.7 µM) PK (189 µM) MET (3.05 µM)	S (124.7 µM) PK (18.9 µM) MET (305.4 nM)	S (12.47 µM) PK (1.89 µM) MET (30.54 nM)	S (1.25 µM) PK (189 nM) MET (3.05 nM)	S (125 nM) PK (18.9 nM) MET (305 pM)
**MIX2**	S (124.7 µM) MET (305.4 µM)	S (124.7 µM) MET (30.54 µM)	S (124.7 µM) MET (3.05 µM)	S (124.7 µM) MET (305.4 nM)	S (12.47 µM) MET (30.54 nM)	S (1.25 µM) MET (3.05 nM)	S (125 nM) MET (305 pM)
**MIX3**	S (124.7 µM) ZOX (463.4 µM) MET (305.4 µM)	S (124.7 µM) ZOX (46.34 µM) MET (30.54 µM)	S (124.7 µM) ZOX (4.63 µM) MET (3.05 µM)	S (124.7 µM) ZOX (463.4 nM) MET (305.4 nM)	S (12.47 µM) ZOX (46.34 nM) MET (30.54 nM)	S (1.25 µM) ZOX (4.63 nM) MET (3.05 nM)	S (125 nM) ZOX (463.4 pM) MET (305 pM)
**MIX4**	S (124.7 µM) PK (18.9 mM) ZOX (463.4 µM) CYF (49.72 µM)	S (124.7 µM) PK (1.89 mM) ZOX (46.34 µM) CYF (4.97 µM)	S (124.7 µM) PK (189 µM) ZOX (4.63 µM) CYF (497.2 nM)	S (124.7 µM) PK (18.9 µM) ZOX (463.4 nM) CYF (49.72 nM)	S (12.47 µM) PK (1.89 µM) ZOX (46.34 nM) CYF (4.97 nM)	S (1.25 µM) PK (189 nM) ZOX (4.63 nM) CYF (497.2 pM)	S (125 nM) PK (18.9 nM) ZOX (463.4 pM) CYF (49.72 pM)
**MIX5**	S (124.7 µM) ZOX (463.4 µM) CYF (49.72 µM)	S (124.7 µM) ZOX (46.34 µM) CYF (4.97 µM)	S (124.7 µM) ZOX (4.63 µM) CYF (497.2 nM)	S (124.7 µM) ZOX (463.4 nM) CYF (49.72 nM)	S (12.47 µM) ZOX (46.34 nM) CYF (4.97 nM)	S (1.25 µM) ZOX (4.63 nM) CYF (497.2 pM)	S (125 nM) ZOX (463.4 pM) CYF (49.72 pM)
**MIX6**		S (124.7 µM) QUI (20.28 µM)	S (124.7 µM) QUI (2.03 µM)	S (124.7 µM) QUI (202.8 nM)	S (12.47 µM) QUI (20.28 nM)	S (1.25 µM) QUI (2.03 nM)	S (125 nM) QUI (202.8 pM)
**MIX7**		PK (1.89 mM) QUI (20.28 µM)	PK (189 µM) QUI (2.03 µM)	PK (18.9 µM) QUI (202.8 nM)	PK (1.89 µM) QUI (20.28 nM)	PK (189 nM) QUI (2.03 nM)	PK (18.9 nM) QUI (202.8 pM)
**MIX8**		S (124.7 µM) MAN (277 µM)	S (124.7 µM) MAN (27.7 µM)	S (124.7 µM) MAN (2.77 µM)	S (12.47 µM) MAN (277 nM)	S (1.25 µM) MAN (27.7 nM)	S (125 nM) MAN (2.77 nM)
**MIX9**	S (124.7 µM) FOL (3.37 mM)	S (124.7 µM) FOL (337 µM)	S (124.7 µM) FOL (33.7 µM)	S (124.7 µM) FOL (3.37 µM)	S (12.47 µM) FOL (337 nM)	S (1.25 µM) FOL (33.7 nM)	S (125 nM) FOL (3.37 nM)
**MIX10**	S (124.7 µM) FOL (3.37 mM) PEN (88 µM)	S (124.7 µM) FOL (337 µM) PEN (8.80 µM)	S (124.7 µM) FOL (33.7 µM) PEN (880 nM)	S (124.7 µM) FOL (3.37 µM) PEN (88 nM)	S (12.47 µM) FOL (337 nM) PEN (8.80 nM)	S (1.25 µM) FOL (33.7 nM) PEN (880 pM)	S (125 nM) FOL (3.37 nM) PEN (88 pM)
**MIX11**		S (124.7 µM) MAN (277 µM) PEN (8.80 µM)	S (124.7 µM) MAN (27.7 µM) PEN (880 nM)	S (124.7 µM) MAN (2.77 µM) PEN (88 nM)	S (12.47 µM) MAN (277 nM) PEN (8.80 nM)	S (1.25 µM) MAN (27.7 nM) PEN (880 pM)	S (125 nM) MAN (2.77 nM) PEN (88 pM)
**MIX12**		S (124.7 µM) MAN (277 µM) MET (30.54 µM)	S (124.7 µM) MAN (27.7 µM) MET (3.05 µM)	S (124.7 µM) MAN (2.77 µM) MET (305.4 nM)	S (12.47 µM) MAN (277 nM) MET (30.54 nM)	S (1.25 µM) MAN (27.7 nM) MET (3.05 nM)	S (125 nM) MAN (2.77 nM) MET (305 pM)
**MIX13**		S (124.7 µM) QUI (20.28 µM) DIM (515.7 µM)	S (124.7 µM) QUI (2.03 µM) DIM (51.57 µM)	S (124.7 µM) QUI (202.8 nM) DIM (5.16 µM)	S (12.47 µM) QUI (20.28 nM) DIM (515.7 nM)	S (1.25 µM) QUI (2.03 nM) DIM (51.57 nM)	S (125 nM) QUI (202.8 pM) DIM (5.17 nM)

Underlining indicates S concentrations present in the same amount (1:1000 dilution) in the 1:1 to 1:1000 mixture dilutions due to low solubility, as described in the Section 2.1 of the Methods.

**Table 2:** Forward and reverse sequences of specific primers used in real-time PCR.

Gene	qPCR Primers (5′-3′)	
**BAX**	fw:	GTCTTTTTCCGAGTGGCAGC
	rev:	GACAGGGACATCAGTCGCTT
**BCL2**	fw:	CTTTGAGTTCGGTGGGGTCA
	rev:	GGGCCGTACAGTTCCACAAA
**NRF2**	fw:	ACAAGATGGGCTGCTGCACTGG
	rev:	TCCCCGAGGAACCCGCTGAAAA
**TBP**	fw:	AACTTCGCTTCCGCTGGCCC
	rev:	ACCCTTGCGCTGGAACTCGT

**Table 3:** BMD_10_ values calculated for metabolic activity (MTS) and cell proliferation (CyQuant) assays in HepG2 and A549 cells with corresponding lower (BMDL) and upper (BMDU) bounds. Hyphens indicate that BMD_10_ values could not be determined due to lack of convergence in the model fit.

	HepG2									A549		
		MTS			CyQuant		MTS			CyQuant		
	BMD_10_	BMDL	BMDU	BMD_10_	BMDL	BMDU	BMD_10_	BMDL	BMDU	BMD_10_	BMDL	BMDU
MIX1	8.26 × 10^−1^	2.08 × 10^−2^	1.19 × 10^0^	2.40 × 10^−3^	1.71 × 10^−6^	2.52 × 10^−3^	-	-	-	1.22 × 10^−3^	1.58 × 10^−5^	0.0216
MIX2	1.06 × 10^−5^	1.00 × 10^−6^	1.14 × 10^−4^	-	-	-	4.19 × 10^−5^	1.33 × 10^−6^	0.00576	1.77 × 10^−2^	9.21 × 10^−4^	3.64 × 10^−1^
MIX3	3.81 × 10^−5^	3.55 × 10^−6^	1.63 × 10^−4^	8.13 × 10^−4^	1.31 × 10^−5^	4.92 × 10^−3^	2.66 × 10^−3^	3.99 × 10^−5^	1.61 × 10^−1^	5.50 × 10^−3^	2.44 × 10^−4^	8.62 × 10^−2^
MIX4	-	-	-	6.22 × 10^−5^	1.03 × 10^−5^	8.84 × 10^−4^	-	-	-	7.36 × 10^−1^	1.77 × 10^−2^	8.29 × 10^−1^
MIX5	4.97 × 10^−6^	1.00 × 10^−6^	1.82 × 10^−4^	1.80 × 10^−4^	1.79 × 10^−5^	4.35 × 10^−4^	-	-	-	-	-	-
MIX6	7.59 × 10^−5^	1.18 × 10^−6^	9.12 × 10^−4^	1.24 × 10^−2^	9.35 × 10^−5^	6.67 × 10^−2^	1.03 × 10^−4^	2.13E-05	2.97 × 10^−4^	1.79 × 10^−2^	1.03 × 10^−3^	1.14 × 10^0^
MIX7	1.11 × 10^−4^	1.67 × 10^−6^	1.98 × 10^−2^	2.36 × 10^−2^	6.22 × 10^−4^	1.15 × 10^−1^	-	-	-	-	-	-
MIX8	7.39 × 10^−4^	2.81 × 10^−4^	2.41 × 10^−3^	5.34 × 10^−3^	2.71 × 10^−3^	1.71 × 10^−2^	4.98 × 10^−2^	2.61 × 10^−3^	5.22 × 10^−2^	5.17 × 10^−2^	1.26 × 10^−2^	5.17 × 10^−2^
MIX9	-	-	-	2.50 × 10^−2^	2.44 × 10^−3^	3.29 × 10^−1^	2.95 × 10^−4^	5.09 × 10^−6^	2.36 × 10^−2^	9.65 × 10^−3^	3.07 × 10^−4^	1.00 × 10^−1^
MIX10	4.74 × 10^−1^	1.23 × 10^−2^	4.80 × 10^−1^	2.19 × 10^−2^	2.71 × 10^−3^	2.03 × 10^−1^	2.54 × 10^−1^	2.21 × 10^−6^	2.21 × 10^−1^	1.61 × 10^−4^	4.03 × 10^−6^	9.45 × 10^−3^
MIX11	3.81 × 10^−4^	2.24 × 10^−4^	1.01 × 10^−3^	8.56 × 10^−4^	2.57 × 10^−4^	3.26 × 10^−3^	7.03 × 10^−3^	4.33 × 10^−3^	1.34 × 10^−2^	1.02 × 10^−3^	1.48 × 10^−4^	5.46 × 10^−3^
MIX12	1.42 × 10^−3^	5.11 × 10^−4^	3.42 × 10^−3^	4.25 × 10^−3^	2.11 × 10^−3^	1.15 × 10^−2^	3.05 × 10^−3^	1.07 × 10^−4^	3.66 × 10^−2^	6.01 × 10^−3^	7.11 × 10^−4^	4.06 × 10^−2^
MIX13	2.66 × 10^−3^	3.02 × 10^−5^	8.53 × 10^−2^	4.52 × 10^−5^	1.32 × 10^−5^	9.88 × 10^−5^	-	-	-	1.59 × 10^−5^	3.73 × 10^−6^	5.24 × 10^−5^

**Table 4:** Toxicological ranking and ToxPI scores of the 13 pesticide mixtures, integrating the overall data (Total) or separated by cell line (HepG2 and A549).

	Total		HepG2		A549	
	Ranking	ToxPi Score	Ranking	ToxPi Score	Ranking	ToxPi Score
MIX11	1	0.7982	1	0.7725	1	0.6461
MIX8	2	0.6784	2	0.7592	4	0.4898
MIX12	3	0.619	3	0.6174	3	0.5218
MIX2	4	0.4936	4	0.4944	2	0.5232
MIX13	5	0.475	5	0.3976	5	0.4710
MIX6	6	0.3675	7	0.3453	6	0.3544
MIX3	7	0.3277	9	0.3359	11	0.2648
MIX4	8	0.2829	8	0.3375	9	0.2837
MIX5	9	0.2642	6	0.3473	12	0.1831
MIX10	10	0.2611	11	0.2704	7	0.3371
MIX9	11	0.2443	12	0.2117	8	0.3321
MIX7	12	0.2231	10	0.3187	13	0.1612
MIX1	13	0.2069	13	0.1897	10	0.2681

## Data Availability

Not applicable.

## Supplementary Materials

The following supporting information can be downloaded at: https://www.mdpi.com/article/10.3390/ijerph19095202/s1. Figure S1: Time-course of apoptosis assessment in (A) HepG2 and (B) A549 cell lines treated with the 13 pesticide mixtures for 24 h at 1:10 (dark blue lines), 1:100 (dark magenta lines) and 1:1000 (dark turquoise lines) field concentrations. Data represent mean fold change luminescence signals, compared to control cells, of three independent experiments. Asterisks indicate the level of significance: * *p* < 0.05; ** *p* < 0.01; *** *p* < 0.001; Figure S2: Time-course of necrosis assessment in (A) HepG2 and (B) A549 cell lines treated with different pesticide mixtures for 24 h at 1:10 (dark blue lines, 1:100 (dark magenta lines) and 1:1000 (dark turquoise lines) field concentrations. Data represent mean fold change fluorescence signals, compared to control cells, of three independent experiments. Among the 13 pesticide mixtures tested, only graphs with significant effects are shown. Asterisks indicate the level of significance: * *p* < 0.05; ** *p* < 0.01; *** *p* < 0.001.
