# Supplementary material for: In Vitro Assessment and Toxicological Prioritization of Pesticide Mixtures at Concentrations Derived from Real Exposure in Occupational Scenarios
Source: Int J Environ Res Public Health. 2022 Apr 25;19(9):5202. doi: 10.3390/ijerph19095202 (PMC9104687; doi:10.3390/ijerph19095202)
Supplement: Supplementary file 1 [file ijerph-19-05202-s001.zip › ijerph-1648692-supplementary.pdf]

A

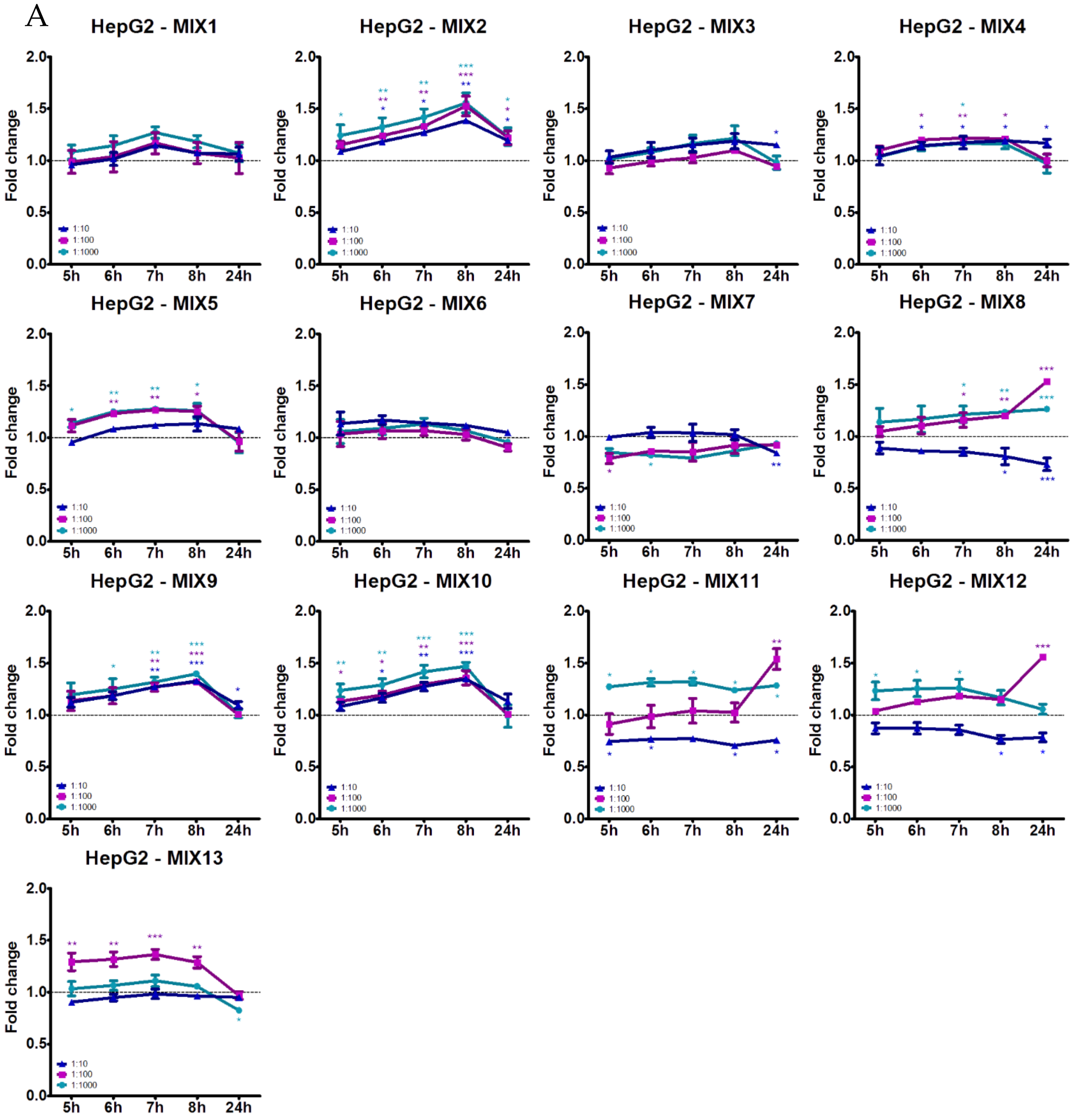
HepG2 - MIX1
HepG2 - MIX2
HepG2 - MIX3
HepG2 - MIX4
HepG2 - MIX5
HepG2 - MIX6
HepG2 - MIX7
HepG2 - MIX8
HepG2 - MIX9
HepG2 - MIX10
HepG2 - MIX11
HepG2 - MIX12
HepG2 - MIX13
Fold change
2.0
1.5
1.0
0.5
0.0
5h
6h
7h
8h
24h
1:10
1:100
1:1000

B

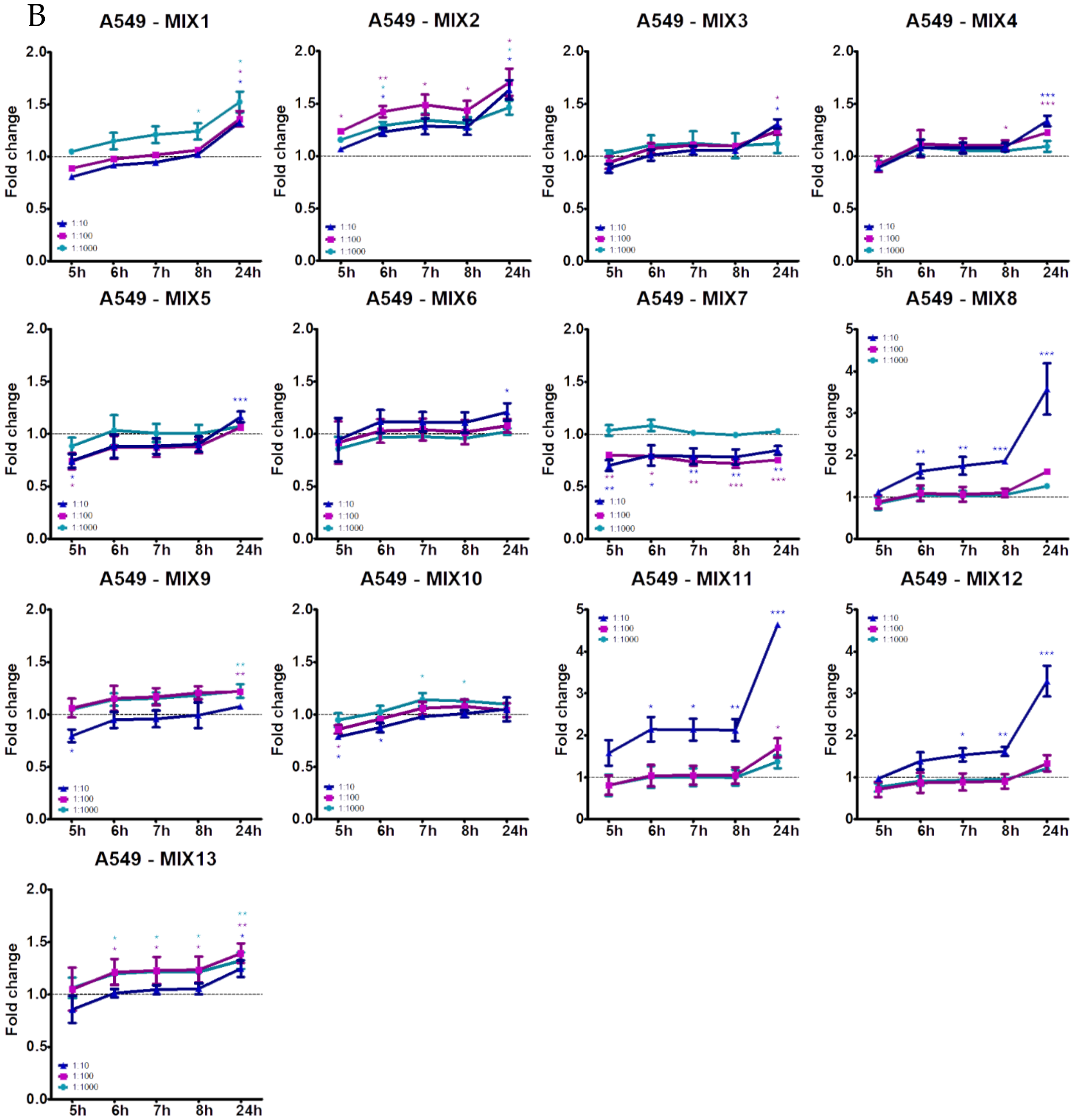

Supplementary Figure S1 – Time-course of apoptosis assessment in A) HepG2 and B) A549 cell lines treated with the 13 pesticide mixtures for 24h at 1:10 (dark blue lines), 1:100 (dark magenta lines) and 1:1000 (dark turquoise lines) field concentrations. Data represent mean fold change luminescence signals, compared to control cells, of three independent experiments. Asterisks indicate the level of significance: *p<0.05; **p<0.01; ***p<0.001.

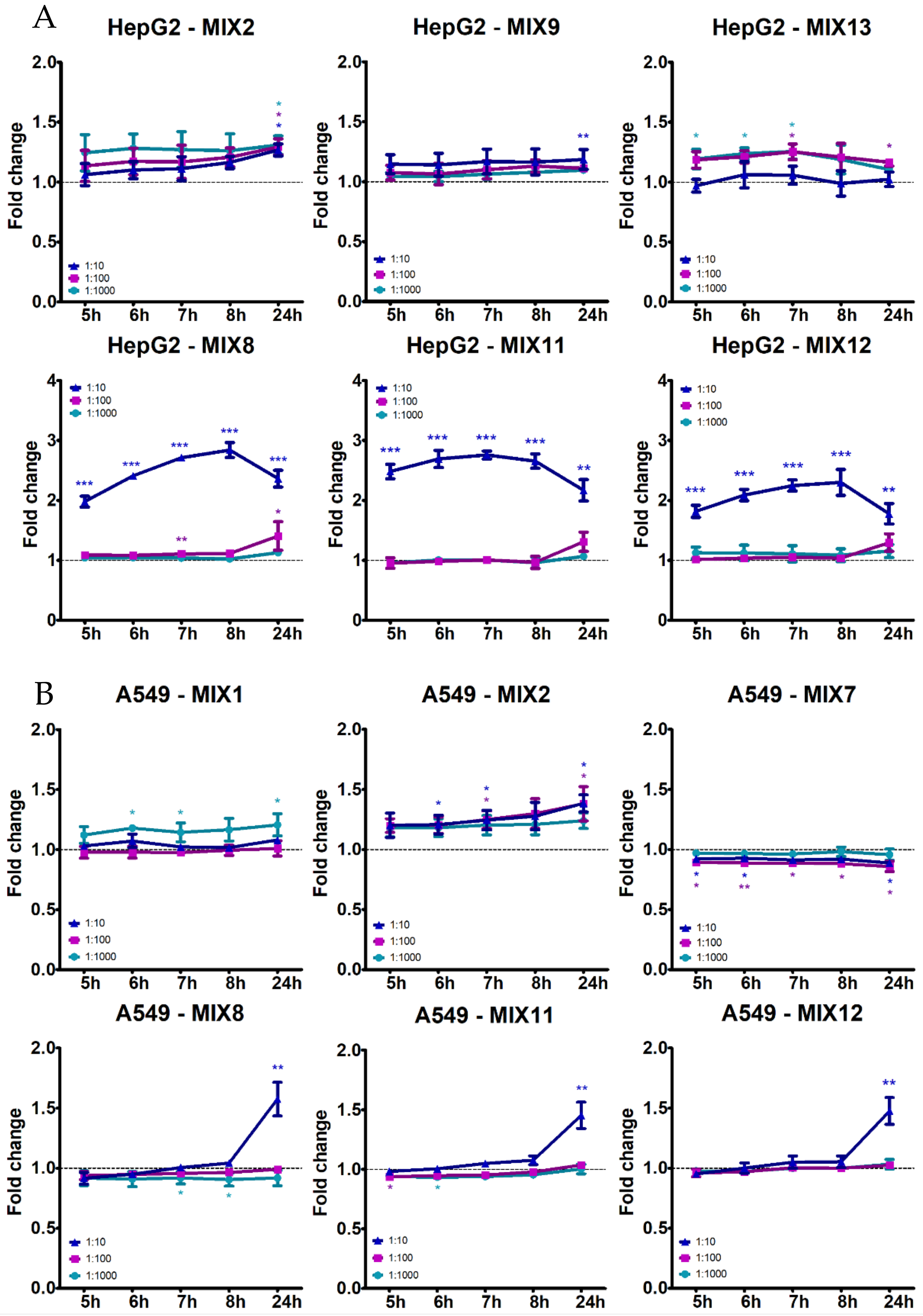

Supplementary Figure S2 – Time-course of necrosis assessment in A) HepG2 and B) A549 cell lines treated with different pesticide mixtures for 24h at 1:10 (dark blue lines, 1:100 (dark magenta lines) and 1:1000 (dark turquoise lines) field concentrations. Data represent mean fold change fluorescence signals, compared to control cells, of three independent experiments. Among the 13 pesticide mixtures tested, only graphs with significant effects are shown. Asterisks indicate the level of significance: *p<0.05; **p<0.01; ***p<0.001.
